# Author Correction: A sulfotransferase dosage-dependently regulates mouthpart polyphenism in the nematode *Pristionchus pacificus*

**DOI:** 10.1038/s41467-018-07354-z

**Published:** 2018-11-13

**Authors:** Linh T. Bui, Nicholas A. Ivers, Erik J. Ragsdale

**Affiliations:** Department of Biology, Indiana University, 915 E. 3rd St., Bloomington, IN 47405 USA

Correction to: *Nature Communications* 10.1038/s41467-018-05612-8, published online 08 October 2018

The original version of this Article contained errors in Figure 4. In panel a, the *x* axis labels of bars 6–11 were incorrect, as depicted below. These errors have been corrected in both the PDF and HTML versions of the Article.

**Figure 1:**
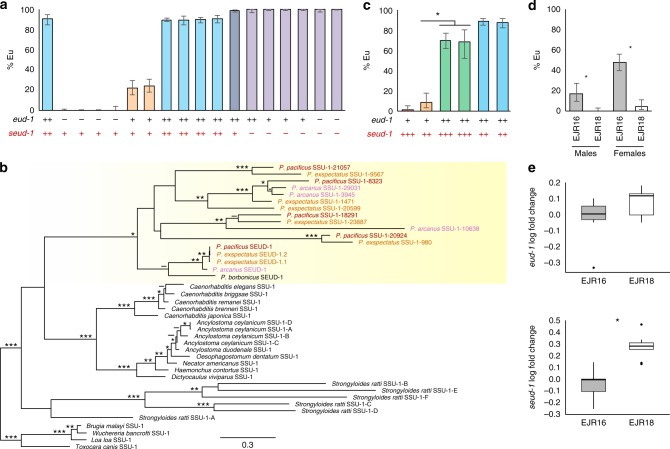
Fig. 4

