# Supplementary material for: A sulfotransferase dosage-dependently regulates mouthpart polyphenism in the nematode Pristionchus pacificus
Source: Nat Commun. 2018 Oct 8;9:4119. doi: 10.1038/s41467-018-05612-8 (PMC6175886; doi:10.1038/s41467-018-05612-8)
Supplement: Supplementary file 1 — Supplementary Information [file 41467_2018_5612_MOESM1_ESM.pdf]

A sulfotransferase dosage-dependently regulates mouthpart polyphenism in the nematode *Pristionchus pacificus*

Bui, Ivers & Ragsdale

Supplementary Information

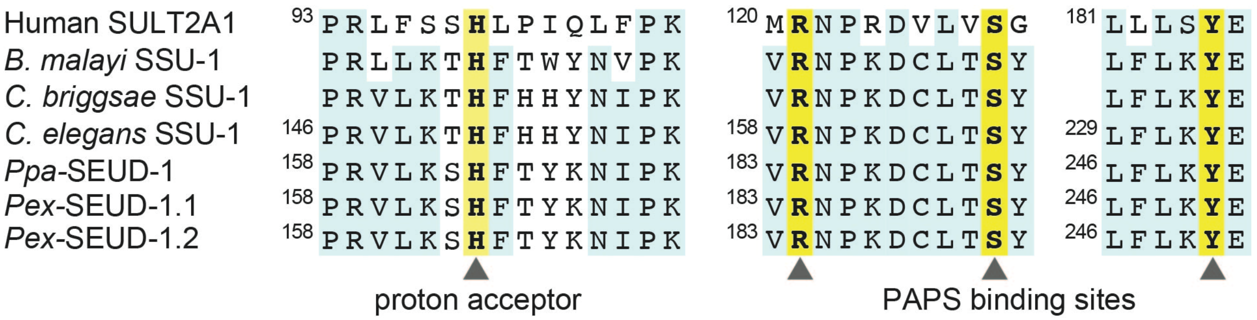

Supplementary Figure 1. Alignment of partial SSU-1 homologues in nematodes and human. Aligned sequences are partial substrate-binding and 3'-phosphoadenosine-5' phosphosulfate (PAPS)-binding domains.

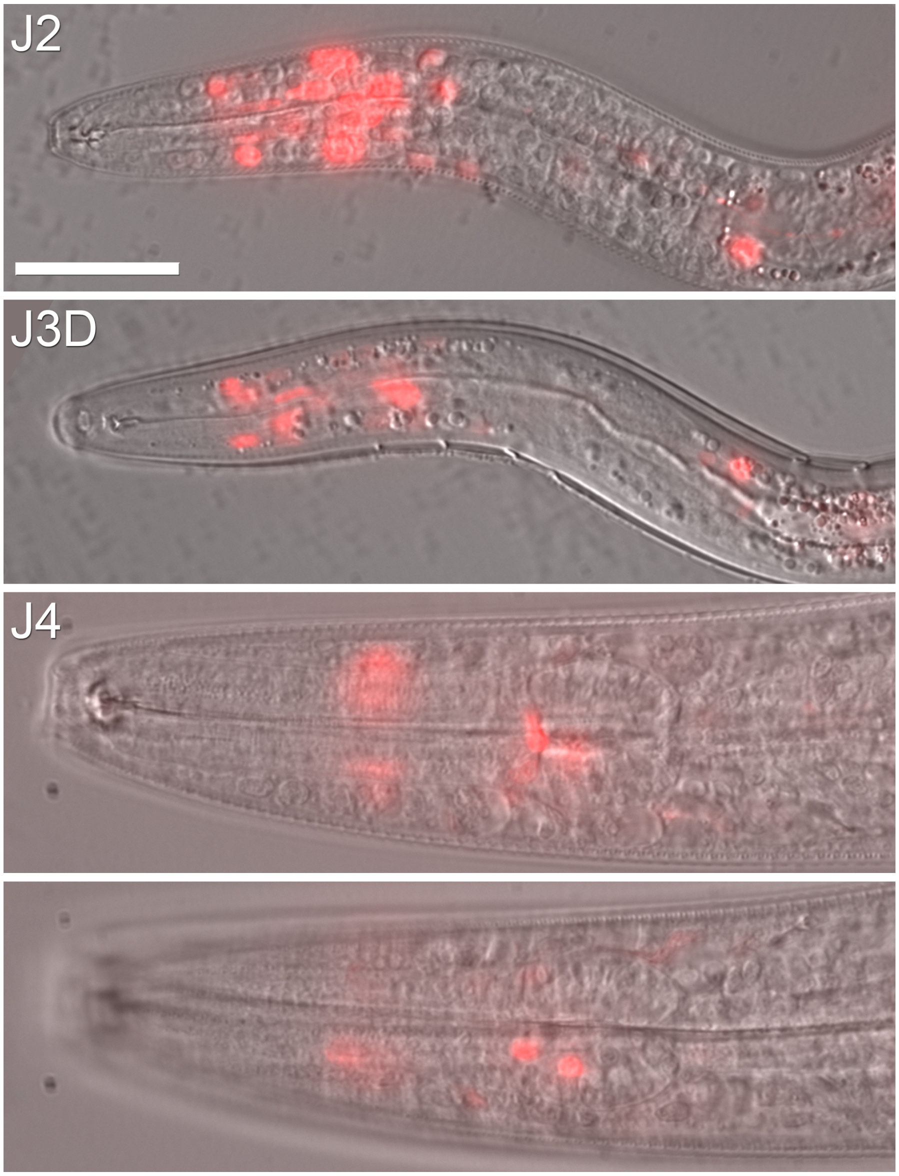

Supplementary Figure 2. Expression of *seud-1* in *Pristionchus pacificus* juveniles. Expression of a nucleus-localised *seud-1*^{promoter}*::TurboRFP* is shown for J2, dauer (J3D), and J4 larvae. The cells that build and make-up dimorphic mouthparts were not all observed to express *seud-1* at all times, including among individuals of a given life-stage, although collectively expression was observed in the same set of cells identified in the text. Pictured J2 shows expression in pharyngeal myoepthelial (pm) and epithelial (mc) cells, as well as in arcade (ac) and epidermal (hyp) cells; dauers reported expression in similar cells, suggesting an overlapping function for *seud-1* in direct-developing and facultative diapause larvae; J4, shown in two focal planes (above, left of sagittal; below, right of sagittal), highlights expression in pm1, pm3, the pharyngeal neuron I1, and possibly I2, another neuron known to innervate teeth-producing (pm1) cells, as well as weak expression in hyp and arcade cells. Scale bar (all images), 20 µm.

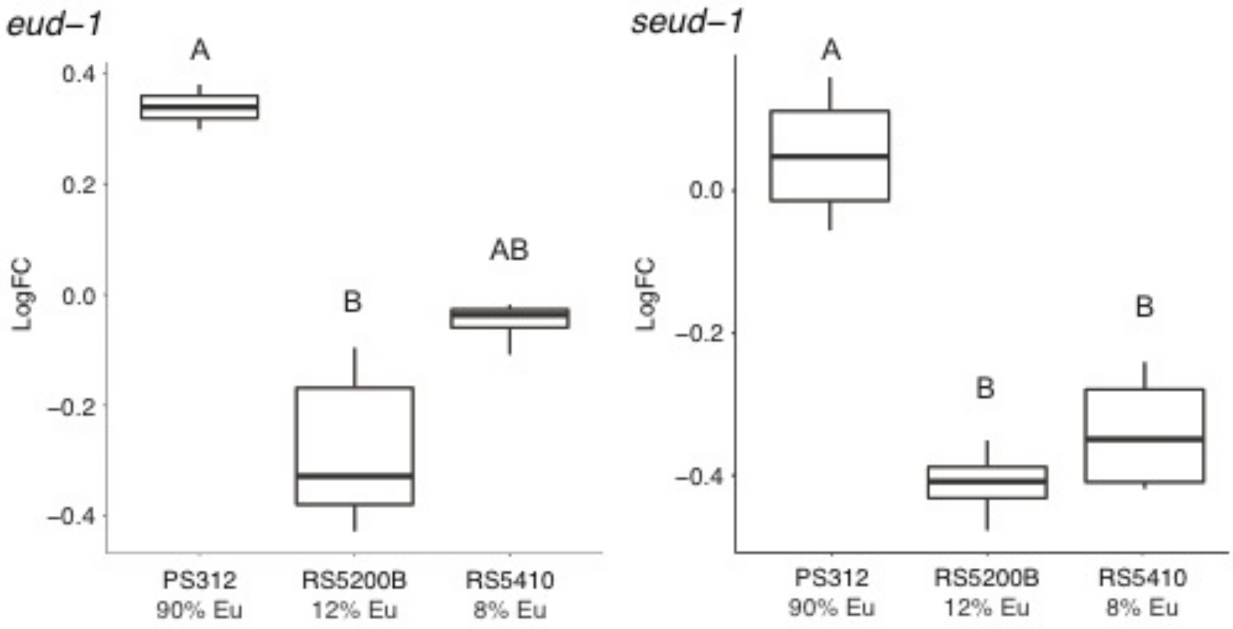

Supplementary Figure 3. Expression levels of *eud-1* and *seud-1* in *Pristionchus pacificus* polyphenism variants. Expression was quantified for wild isolates with different morph-ratios under standardised environmental cues. Mean expression levels of *eud-1* and *seud-1* were calculated as log fold change (LogFC) normalized to two reference genes (*ttb-4* and Y45F10D). We found significant differences among strains in *eud-1* expression (P = 0.03, Tukey's test). Specifically, the Eu-biased California strain (PS312) had significantly higher expression of *eud-1* than the St-biased strain RS5200B (P_{adj} = 0.03, Tukey's test) and had a non-significant trend for higher expression than the St-biased strain RS5410 (P_{adj} = 0.10, Tukey's test). Additionally, we found significant differences among strains in s*eud-1* expression (P = 0.02, Tukey's test). Here, the reference strain had a significantly higher level of *seud-1* expression than two of the other strains (RS5200B and RS5410; P = 0.02 and P = 0.03, respectively, Tukey's test). % Eu, mean proportion of eurystomatous nematodes under standardised laboratory conditions, as previously reported[1]. Box plots show ΔΔCt values (centre line, median; box limits, upper and lower quartiles; whiskers, 1.5x interquartile range).

Supplementary Table 1. Mouth-morph phenotypes for reference and mutant strains of *Pristionchus pacificus*.

Genotype	% Eu	*n*
PS312	90	250
eud-1(tu445)	0	250
seud-1(iub7); eud-1(tu445)	100	260
seud-1(iub8); eud-1(tu445)	100	120

Supplementary Table 2. Summary of mutant lesions of *seud-1* alleles.

Allele	Lesion	Genome position	Location in gene	Effect
iub7	G to A	Contig20:1004979	Exon 8	STOP
iub8	C to T	Contig20:1009019	Exon 1	STOP

Genome position is given with respect to the “Hybrid1” assembly for *Pristionchus pacificus.*

Supplementary Table 3. Primers used to confirm *seud-1* and *eud-1* genotypes.

Gene	Forward primer	Reverse primer	Amplicon length (bp)
seud-1	GAAAGAGAATGCGCTCTTCG	GGATGGAAAGGTGGGTTTCT	338
eud-1	TCACTACCGGAGGGTGCTAC	TTCTACGTTTCGCTCTGCAA	361

Amplicons distinguished between mutant (*iub7*, *tu445*) and wild-type genotypes following outcrossing and generation of double- and triple-mutant lines of *Pristionchus pacificus*.

Supplementary Table 4. Mouth-morph phenotypes for rescued strains and epistasis tests.

Genotype	% Eu	*n*
Ex[*seud-1*]	28	212
nhr-40(tu505)	100	250
nhr-40(tu505); Ex[*seud-1*]	100	262
Ex[*nhr-40*]	1	139
seud-1(iub7); Ex[*nhr-40*]	100	61

Supplementary Table 5. Primers used for generating transgenic *seud-1* constructs.

Region	Forward primer	Reverse primer	Amplicon length (bp)
Promoter	TCTGAAGGGGTACGGTAGG	TCACGGATCCAACCTTTTTCGA	4,223
Full gene	CTTTATCAACGAATTAGTAGGAA	CAGCAACTTTCACTACGTGTCTG	10,983

Supplementary Table 6. Crosses performed to manipulate gene dosage in *Pristionchus pacificus*.

Cross type	Crosses (*N*)	F1 genotypes	% Eu	*n*
PS312 ♂ x *pdl-2(-)* ⚥	28	*seud-1(+/+); eud-1(+/+)*	24	160
		seud-1(+/+); eud-1(+/0)	91	198
seud-1(iub7); eud-1(tu445) ♂ x *pdl-2(-); eud-1(tu445)* ⚥	30	*seud-1(+/-); eud-1(-/-)*	0	248
		seud-1(+/-); eud-1(-/0) #2	0	708
PS312 ♂ x *pdl-2(-); eud-1(tu445)* ⚥	45	*seud-1(+/+); eud-1(-/+)*	22	148
		seud-1(+/+); eud-1(-/0)	0	25
seud-1(iub7) ♂ x *pdl-2(-); eud-1(tu445)* ⚥	90	*seud-1(+/-); eud-1(-/+)*	90	1231
		seud-1(+/-); eud-1(-/0) #1	0	1988
seud-1(iub7); eud-1(tu445) ♂ x *pdl-2(-)* ⚥	27	*seud-1(+/-); eud-1(+/-)*	90	202
		seud-1(+/-); eud-1(+/0) #1	90	556
seud-1(iub7) ♂ x *pdl-2(-)* ⚥	26	*seud-1(+/-); eud-1(+/+)*	99	406
		seud-1(+/-); eud-1(+/0) #2	91	289
seud-1(iub7); eud-1(tu445) ♂ x *seud-1(iub7); pdl-2(-)* ⚥	14	*seud-1(-/-); eud-1(+/-)*	100	127
		seud-1(-/-); eud-1(+/0) #1	100	298
seud-1(iub7) ♂ x *seud-1(iub7); pdl-2(-)* ⚥	26	*seud-1(-/-); eud-1(+/+)*	100	260
		seud-1(-/-); eud-1(+/0) #2	100	437
seud-1(iub7); eud-1(tu445) ♂ x *seud-1(iub7); eud-1(tu445)* ⚥	7	*seud-1(-/-); eud-1(-/-)*	100	509
		seud-1(-/-); eud-1(-/0)	100	146

Supplementary Table 7. *seud-1* homologues extracted from published genome sequences.

Species	Gene or putative gene	Sequence identifier	Transcript
Ancylostoma ceylanicum	*ssu-1-A*	ANCCEY_10587	Predicted
Ancylostoma ceylanicum	*ssu-1-B*	EYC21177	Predicted
Ancylostoma ceylanicum	*ssu-1-C*	EYC21178	Predicted
Ancylostoma ceylanicum	*ssu-1-D*	EYC21179.1	Predicted
Ancylostoma duodenale	*ssu-1*	ANCDUO_03922	Predicted
Brugia malayi	*ssu-1*	Bm7300a	Partially confirmed
Caenorhabditis brenneri	*ssu-1*	CBN06215	Predicted
Caenorhabditis briggsae	*ssu-1*	CBG12932	Confirmed
Caenorhabditis elegans	*ssu-1*	CELE_Y113G7A.11	Partially confirmed
Caenorhabditis japonica	*ssu-1*	CJA13369n	Predicted
Caenorhabditis remanei	*ssu-1*	CRE21191	Predicted
Dictyocaulus viviparus	*ssu-1*	DICVIV07955	Predicted
Haemonchus contorus	*ssu-1*	HCOI_00221200	Predicted
Loa loa	*ssu-1*	LOAG_07209	Predicted
Necator americanus	*ssu-1*	NECAME_16579	Predicted
Oesophagostomum denatum	*ssu-1*	OESDEN_03346	Predicted
Pristionchus borbonicus	*seud-1*	scaffold4422_c61143	Confirmed
Pristionchus exspectatus	*seud-1.1*	scaffold2136-pex_aug2013-10260	Confirmed
Pristionchus exspectatus	*seud-1.2*	scaffold86-pex_aug2013-27432	Confirmed
Pristionchus exspectatus	*ssu-1* homologue 980	scaffold1056-pex_aug2013-980	Predicted
Pristionchus exspectatus	*ssu-1* homologue 1471	scaffold1098-pex_aug2013-1471	Predicted
Pristionchus exspectatus	*ssu-1* homologue 9567	scaffold201-pex_aug2013-9567	Confirmed
Pristionchus exspectatus	*ssu-1* homologue 10260	scaffold2136-pex_aug2013-10260	Confirmed
Pristionchus exspectatus	*ssu-1* homologue 20599	scaffold494-pex_aug2013-20599	Confirmed
Pristionchus exspectatus	*ssu-1* homologue 23887	scaffold64-pex_aug2013-23887	Confirmed
Pristionchus exspectatus	*ssu-1* homologue 27432	scaffold86-pex_aug2013-27432	Confirmed
Pristionchus pacificus	*seud-1*	Contig20-aug8366.t1	Confirmed
Pristionchus pacificus	*ssu-1* homologue 8323	Contig20-aug8323.t1	Confirmed
Pristionchus pacificus	*ssu-1* homologue 18291	Contig80-aug18291.t1	Predicted
Pristionchus pacificus	*ssu-1* homologue 20924	Contig113-aug20924.t1	Partially confirmed
Pristionchus pacificus	*ssu-1* homologue 21057	Contig116-aug21057.t1	Confirmed
Strongyloides ratti	*ssu-1-A*	SRAE_0000037000	Partially confirmed
Strongyloides ratti	*ssu-1-B*	SRAE_2000472200	Predicted
Strongyloides ratti	*ssu-1-C*	SRAE_1000077400	Predicted
Strongyloides ratti	*ssu-1-D*	SRAE_2000454300	Predicted
Strongyloides ratti	*ssu-1-E*	SRAE_2000472300	Predicted
Strongyloides ratti	*ssu-1-F*	SRAE_2000472100	Predicted
Toxocara canis	*ssu-1*	Tcan_04678	Predicted
Wucheria bancrofti	*ssu-1*	WUBG_09659	Predicted

Supplementary Table 8. Primers to quantify *eud-1* and *seud-1* expression in *Pristionchus exspectatus* inbred lines.

Gene	Forward primer	Reverse primer	Amplicon length (bp)
eud-1	ATGTCATCATACTCATGGTGGAC	CTCCGTCAGTACTGGTTTCTTCAT	210
seud-1A	CTCGTGGTTGGAGGGCATCGAAA	CCGCCTAAGAACGAGGCAATTTGA	114
seud-1B	ATCAGTTTGGATACGAACAATCCT	TATGAAGACACTTCACTGGTTGGT	154
tbb-4 (reference)*	CTCGGAGGAGGAACTGGATC	GACCGTGTCAGAGACCTTAG	123

*Primer sequence published previously[2].

Supplementary Table 9. Genotypes and phenotypes of interspecific hybrids.

Cross type	F1 genotype	% Eu	Crosses (*N*)	n
RS5522B ♂ x *pdl-2(-); eud-1(tu445)* ⚥	*eud-1(-/+); seud-1(+/++)*	1	14	114
seud-1(iub7); eud-1(tu445) ♂ x RS5522B ♀	*eud-1(+/-); seud-1(++/-)*	8	19	72
PS312 ♂ x RS5522B ♀	*eud-1(+/+); seud-1(++/+)*	70	15	153
RS5522B ♂ x *pdl-2(-)* ⚥	*eud-1(+/+); seud-1(+/++)*	68	21	47
seud-1(iub7) ♂ x RS5522B ♀	*eud-1(+/+); seud-1(++/-)*	89	54	404
RS5522B ♂ x *pdl-2(-); seud-1(iub7)* ⚥	*eud-1(+/+); seud-1(++/-)*	87	34	227

PS312, wild-type *P. pacificus*; RS5522B, *P. exspectatus*.

Supplementary Table 10. Primers to quantify *seud-1* and *eud-1* expression in *Pristionchus pacificus* isolates.

Gene	Forward primer	Reverse primer	Amplicon length (bp)
eud-1	GGCACATTCGACCTCATGTCGA	TGCCGCCTGGACACATCTGCT	157
seud-1	AAGTACATTTTTGCAGTGAGAA	TGAACAACTTGAAGAACACATC	121
tbb-4 (reference)*	CTCGGAGGAGGAACTGGATC	GACCGTGTCAGAGACCTTAG	123
Y45F10D (reference)*	GATCAGAGTCGACGATAATG	CTTCTCAGCCCATTCGATG	130

*Primer sequences published previously[2].

Supplementary references

1. Ragsdale, E. J., Müller, M. R., Rödelsperger, C. & Sommer, R. J. A developmental switch coupled to the evolution of plasticity acts through a sulfatase. *Cell* **155,** 922–933 (2013).
2. Schuster, L. N. & Sommer, R. J. Expressional and functional variation of horizontally acquired cellulases in the nematode *Pristionchus pacificus*. *Gene* **506,** 274–282 (2012).
